# Using iPS Cells toward the Understanding of Parkinson’s Disease

**DOI:** 10.3390/jcm4040548

**Published:** 2015-03-30

**Authors:** Roger Torrent, Francesca De Angelis Rigotti, Patrizia Dell’Era, Maurizio Memo, Angel Raya, Antonella Consiglio

**Affiliations:** 1 Institute for Biomedicine of the University of Barcelona (IBUB), Barcelona Science Park, Barcelona 08028, Spain; E-Mails: rtorrent@gmail.com (R.T.); fdeangelisrigotti@ibub.pcb.ub.es (F.D.A.R.); 2 Department of Molecular and Translational Medicine, Fibroblast Reprogramming Unit, University of Brescia, Brescia 25123, Italy; E-Mails: patrizia.dellera@med.unibs.it (P.D.E.); maurizio.memo@med.unibs.it (M.M.); 3 Control of Stem Cell Potency Group, Institute for Bioengineering of Catalonia (IBEC), Barcelona 08028, Spain; E-Mail: araya@ibecbarcelona.eu; 4 Institució Catalana de Recerca i Estudis Avançats (ICREA), Barcelona 08010, Spain; 5 Center for Networked Biomedical Research on Bioengineering, Biomaterials and Nanomedicine (CIBER-BBN), Madrid 28029, Spain; 6 Center of Regenerative Medicine in Barcelona, Dr. Aiguader 88, Barcelona 08003, Spain

**Keywords:** induced pluripotent stem cells, Parkinson’s disease, Leucine-rich repeat kinase 2 (LRRK2), dopaminergic neurons

## Abstract

Cellular reprogramming of somatic cells to human pluripotent stem cells (iPSC) represents an efficient tool for *in vitro* modeling of human brain diseases and provides an innovative opportunity in the identification of new therapeutic drugs. Patient-specific iPSC can be differentiated into disease-relevant cell types, including neurons, carrying the genetic background of the donor and enabling *de novo* generation of human models of genetically complex disorders. Parkinson’s disease (PD) is the second most common age-related progressive neurodegenerative disease, which is mainly characterized by nigrostriatal dopaminergic (DA) neuron degeneration and synaptic dysfunction. Recently, the generation of disease-specific iPSC from patients suffering from PD has unveiled a recapitulation of disease-related cell phenotypes, such as abnormal α-synuclein accumulation and alterations in autophagy machinery. The use of patient-specific iPSC has a remarkable potential to uncover novel insights of the disease pathogenesis, which in turn will open new avenues for clinical intervention. This review explores the current Parkinson’s disease iPSC-based models highlighting their role in the discovery of new drugs, as well as discussing the most challenging limitations iPSC-models face today.

## 1. Parkinson’s Disease

Parkinson’s disease (PD) is the second most common neurodegenerative disease in the world after Alzheimer’s disease (AD), affecting 2% of the population over the age of 60. The mean duration of the disease from the time of diagnosis to death is approximately 15 years, with a mortality ratio of 2 to 1 in the affected subjects [1].

PD is characterized by debilitating motor deficits, such as tremor, limb rigidity and slowness of movements (bradykinesia) although non-motor features, such as hyposmia, cognitive decline, depression, and disturbed sleep are also present in later stages of the disease [1,2,3]. Neuropathologically, these motor deficits are caused by the progressive preferential loss of striatal-projecting neurons of the substantia nigra pars compacta; more specifically a subtype of dopaminergic neurons (DAn) patterned for the ventral midbrain (vmDAn). Neuronal loss is typically accompanied by the presence of intra-cytoplasmic ubiquitin-positive inclusions in surviving neurons. These structures are known as Lewy bodies and Lewy neurites and they are mainly composed of the neuronal protein α-synuclein (α-syn). These protein inclusions are not only found throughout the brain but also outside of the CNS. Moreover, microglial activation and an increase in astroglia and lymphocyte infiltration also occur in PD [4].

Approximately 90%–95% of all PD cases are sporadic with no family history. Although disease onset and age are highly correlated, PD occurs when complex mechanisms such as mitochondrial activity, autophagy or degradation via proteasome are dysregulated by environmental influence or PD-specific mutation susceptibility [5].

Studies of rare large families showing classical Mendelian inherited PD have allowed for the identification of 11 genes out of 16 identified disease *loci*. They include dominant mutations in Leucine-rich repeat kinase 2 (*LRRK2*), recessive mutations in Parkin (coded by *PARK2*) and PTEN-induced putative kinase (*PINK1*) [6], as well as both rare dominant mutations and multiplications in the gene encoding α-synuclein (*SNCA*).

Current treatment for PD is limited to targeting only the symptoms of the disease and does not cure or delay disease progression. Therefore, the identification of new and more effective drugs to slow down, stop and even reverse PD is critical. This limited symptomatic treatment is due to the lack of clear understanding of the underlying mechanisms affected during PD. Using patient-specific iPSC-based models to recapitulate the disease from start to finish delivers a more detailed picture of the mechanisms involved in the progression of Parkinson’s disease and will aid in the discovery of disease-targeted therapies in the future.

## 2. Models of Parkinson’s Disease

Despite advances in the identification of genes and proteins involved in PD, there are still gaps in our understanding of the underlying mechanisms involved [7,8]. The lack of PD models fully representing the complex mechanisms involved in disease progression, as well as the near impossible task of extracting live neurons from patients has proven the investigation of PD difficult [8]. In general, genetic mouse models do not represent the pathophysiological neurodegeneration and protein aggregation pattern observed in PD patients [9,10], and are thus limited [11,12]. On the other hand, PD animal models of administration of neurotoxins systemically or locally have successfully replicated DAn neurodegeneration, however they fail to recapitulate the degeneration in a slow and progressive manner, nor the formation of Lewy body-like inclusions which occur in PD human pathology [13].

Although the cellular models of PD, mostly based on human neuronal tumor cell lines, have provided helpful insights into alterations in specific subcellular components (such as proteasome, lysosome and mitochondrion), the relevance of these findings for PD pathogenesis is not always immediate. These models do not, however, investigate the defective mechanisms within the predominantly affected cell in PD, the DAn [14]. In addition, all studies involving human tissue have been performed with post-mortem samples, which can only allow for a limited analysis.

**Figure 1:**
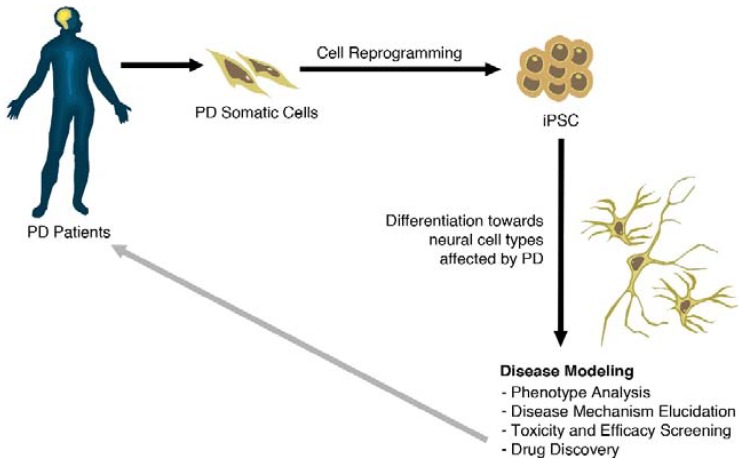
Generation and use of iPSC modelling in PD. Somatic cells from a diseased patient are isolated and then reprogrammed to a pluripotent state (iPSCs). iPSCs can be maintained in culture or induced to differentiate along tissue- and cell-type specific pathways. Differentiated cells can be used to elucidate disease mechanism pathways, as well as for the development of novel therapies.

The recent discovery of cellular reprogramming to generate induced pluripotent stem cells (iPSC) from patient somatic cells offers a remarkable opportunity to generate disease-specific iPSC [15], and to reproduce at a cellular and molecular level the mechanisms involved in disease progression. The use of iPSC offers not only the possibility of addressing important questions such as the functional relevance of the molecular findings, the contribution of individual genetic variations, patient-specific response to specific interventions, but also helps to recapitulate the prolonged time-course of the disease (Figure 1).

## 3. Generation of PD-Specific iPSCs

In recent years, neurodegenerative disease research has quickly advanced with the help of stem cell technology reprogramming somatic cells, such as fibroblasts, into induced pluripotent stem cells (iPSC) [15]. Human iPSC share many characteristics with human embryonic stem cells (hESC), including similarities in their morphologies, gene expression profiles, self-renewal ability, and capacity to differentiate into cell types of the three embryonic germ layers *in vitro* and *in vivo* [16]. An important advantage of induced cell reprogramming is represented by the possibility of generating iPSC from patients showing sporadic or familial forms of the disease. These *in vitro* models are composed of cells that carry the patients’ genetic variants, some known and others not, that are key to the contribution of disease onset and progression. Moreover, given that iPSC can be further differentiated into neurons, this technology potentially provides, for the first time, an unlimited source of native phenotypes of cells specifically involved in the process related to neuronal death in neurodegeneration *in vitro.*

One issue found in modeling PD with the use of iPSC is to correctly reproduce its late-onset characteristics, since aging is a crucial risk factor. Indeed, at first it was unclear whether disease-specific features of neurodegenerative disorders that usually progressively appear over several years were reproducible *in vitro* over a period of only a few days to a few months. As a consequence, iPSC were initially used to model neurodevelopmental phenotypes and a variety of monogenic early-onset diseases [17,18,19,20,21,22,23,24]. However, studies using iPSC derived from patients with monogenic and sporadic forms of PD have illustrated these key features of PD pathophysiology, as a late-onset neurodegenerative disorder, after differentiating these iPSC into dopaminergic neurons. Moreover, several inducible factors that cause cell stress, such as mitochondrial toxins [25], growth factor deficiency, or even modulated aging with induced expression of progerin (a protein causing premature aging) [26], have also been used to accelerate and reproduce the phenotypes found during disease progression.

In this review, the recent work on iPSC-based PD modeling for both sporadic and familial cases will be discussed, as well as how iPSC-based studies are helping in the advancement of novel drug discoveries. These studies give insight for the fundamental understanding of PD pathogenesis, which is critical for the development of new treatments.

## 4. Modeling Sporadic and Familial PD Using iPSC

Over the last few years, several studies have reported the generation of iPSC from patients suffering from sporadic and genetic forms of PD (Table 1). The first group generated PD-specific iPSC from a sporadic PD patient in 2008 [27]. Over the following year, the Jaenisch’s group was able to demonstrate that iPSC derived from PD patients were able to differentiate towards DAn, however, no characteristic signs of progressive neurodegeneration or disease-related phenotypes were observed in those cells [28]. The Jaenisch group generated gene-free iPSC lines from skin fibroblasts of five idiopathic PD patients. Using *in vivo* experiments, they showed that PD-specific iPSC-derived DAn were able to survive and engraft in the rodent striatum for at least 12 weeks. A small number of these cells co-expressed tyrosine hydroxylase (TH) and G-protein-gated inwardly rectifying K+ channel subunit (GIRK2), which are the hallmark characteristics of vmDAn. Remarkably, injection of these iPSC-derived DAn into the brains of 6-OHDA-lesioned rats resulted in motor symptoms improvement [29].

**Table 1:** Summary of the described PD iPSC modeling publications in this review.

Gene	Publication	Mutation	Number of patients	Isogenic Controls	Cell Type Differentiation	Findings
*SNCA*	Devine *et al*., 2011 [30]	Triplication	1	NO	Floor-plate DAn differentiation (21–30 days): 28%–37% TH^+^/TUJ1^+^	mRNA doubled expression of SNCA
	Byers *et al*., 2011 [31]	Triplication	1	NO	DAn differentiation (50 days): 6%–11% TH^+^	Double expression of SNCA, increased susceptibility to OS
	Chung *et al*., 2013 [32]	A53T	2	YES	Neuronal differentiation (56–84 days): DAn yield not specified.	Increased nitrosative stress, and ER stress, reversed by adding NAB2.
	Ryan *et al*., 2013 [25]	A53T	1	YES	Kriks’s Floor-plate DAn differentiation: ~80% A9 DAn of total neurons.	Diminished spare respiration mitochondrial capacity; increased ROS/RNS and attenuation of MEF2/PGC1α neuroprotective pathway
*GBA1*	Mazzulli *et al*., 2011 [33]	N370S/84GG insertion	1	NO	DAn diff. (30 days): 80% TUJ1^+^, 10% TH^+^/TUJ1^+^	Formation of soluble α-syn oligomers, correlated with a decline of lysosomal proteolysis.
	Schöndorf *et al*., 2014 [34]	GBA1 (RecNcil/wt) GD (N370S; L444P)	4 GBA1 4 GD	YES	Kriks’s Floor-plate DAn differentiation: 15%–20% TH^+^/GIRK2^+^/FOXA2^+^/VMAT2^+^ There is also further purification of DAn by FACS	Causal relation of GBA1 mutations with increased a-syn and LB inclusions, correlated with autophagic/lysosomal system impairment
*PARK2*	Jiang *et al*., 2012 [35]	Exon 3/5 deletion	2	NO	DAn differentiation (70 days): yield not specified	Loss of Parkin function; decreased DA uptake and incorrectly folded DAT protein, with increased OS susceptibility. Transduction of WT PARK2 reversed OS sensitiveness.
	Imaizumi *et al*., 2012 [36]	Exons 2–4 and 6,7 homozygous deletion	2	NO	DAn differentiation (10 days): yield not specified	Abnormal mitochondrial morphology and impaired mitochondrial homeostasis.
*PARK2 PINK1*	Miller *et al*., 2013 [26]	PINK1 (Q456X) Parkin (V324a)	1 1	NO	Kriks’s Floor-plate DAn differentiation yield not specified	Loss of dendrite lenght and decreased neuronal survival, as seen by decreased *p*-ATK values, when exposing mDA neurons to progerin.
*PINK1*	Seibler *et al*., 2013 [37]	C1366T, C509G	3	NO	Floor-plate DAn differentiation: 11%–16% TH^+^/TUJ1^+^	Endogenous mutant PINK1 diminished Parkin recruitment to the mitochondrial membrane under the presence of valynomycin. WT PINK1 rescued Parkin recruitment.
*(PINK1)*	Cooper *et al*., 2012 [38]	Q456X	2	NO	DAn differentiation (22 days): 35% TUJ1^+^; 10% TH^+^	Increased vulnerability of neural cells to chemical stressors, with common defects to protect against OS.
*LRRK2*	Nguyen *et al*., 2011 [39]	G2019S, R1441C	2	NO	Floor-plate DAn differentiation (30–35 days): 3.6%–5% TH^+^	α-syn accumulation, increased OS genes, and increased susceptibility to hydrogen peroxide.
	Sánchez-Danes *et al*., 2012 [40]	G2019S	7 Sporadic 4 LRRK2 (G2019S)	NO	DAn diff (Lentiviral-mediated forced expression LMX1A in neural precursors) (75 days): 55% TH^+^/TUJ1^+^ (Majority TH^+^GIRK2^+^)	Reduced neurite lenght and number. Accumulation of α-syn in LRRK2 DAn. Reduction of autophagic flux and accumulation of early autophagosomes.
	Orenstein *et al*., 2013 [41]	G2019S	4 LRRK2 (G2019S)	NO	As described in [40]	Blockage of the CMA degradation pathway due to accumulated α-syn with correlated increased expression of LAMP-2A.
	Reinhardt *et al*., 2013 [42]	G2019S	2	YES	Floor-plate DAn differentiation (30–35 days): 20% TH/TUJ1/DAPI	Decreased neurite lenght levels. Increased ERK activation levels, and discover of novel genes dysregulated in LRRK2 DAn.

Many laboratories have now successfully recapitulated *in vitro* some of the characteristics of PD, using iPSC as a model compared to the aforementioned studies in which no signs of Parkinson’s disease were observed. However, given that PD is a progressive aging disease that affects several cellular mechanisms involving different cell types, each iPSC model highlights only some PD-associated characteristics. Nevertheless, each one of these models has helped to understand some of the fundamental underlying mechanisms as a proof-of-concept. In the last few years, iPSC-model reliability has rapidly improved and has paved the way for the discovery of new complex biomolecular interactions in the pathogenesis of PD. Thus, iPSC modeling has shown to be promising as a tool for drug-screening platforms in the future.

Recently, iPSC-derived DA neurons carrying a triplication of *SNCA*, the coding gene for α-syn protein, have been generated [30,31]. These cells showed enhanced α-syn mRNA and protein levels [30] and increased cell death vulnerability when exposed to oxidative-stress inducers [31]. Using an iPSC model based on the rare missense A53T *SNCA* mutation, Chung *et al*. observed early pathogenic phenotype in patient-derived neurons, compared to isogenic gene-corrected controls. In particular, they observed a connection between nitrosative and ER stress in the context of α-syn toxicity. Interestingly, the levels of CHOP (CCAAT enhancer binding protein homologous protein), a component of ER stress-induced apoptosis, did not change, indicating that in this model cellular pathology was still at an early stage [32]. iPSC-derived DAn, carrying the A53T *SNCA* mutation, also showed α-syn aggregation, altered mitochondrial machinery, thus enhancing basal ROS/RNS production [25]. The increase of RNS production leads to *S*-nitrosylation of the *pro*-survival transcription factor MEF2 and its consequent inhibition, reducing the expression of the mitochondrial master regulator PGC1α and genes that are important for the development and survival of A9 DAn [43]. Interestingly, Ryan *et al*., postulated that the MEF2-PGC1α pathway contributes to the appearance of late-onset phenotypes in PD due to the complex interaction between environmental factors and gene expression. Indeed, when PD-associated pesticides were added below EPA-accepted levels, this was enough to exacerbate oxidative/nitrosative stress, inhibiting MEF2-PGC1α and inducing apoptosis, a late-onset phenotype [25].

Interestingly, α-syn is one of the main pathological readouts for many of the sporadic and familial PD cases that are not related with mutations in *SNCA* [44]. For example, the clinical link between the lysosomal storage disorder Gaucher disease (GD) and PD appears to be based on the fact that mutations in acid *GBA1* gene, which causes GD, contributes to the pathogenesis of synucleinopathies [33,34]. *GBA1* encodes the lysosomal enzyme β-Glucocerebrocidase (GCase), which cleaves the β-glucosyl linkage of GlcCer. Functional loss of GCase activity in iPSC-derived neurons has been associated with compromised lysosomal protein degradation, which in turn induces α-syn accumulation, resulting in neurotoxicity through aggregation-dependent mechanisms [33]. In addition, iPSC-derived neurons carrying the heterozygous mutation in *GBA1* also have shown increased levels of GlcCer, changes in the autophagic/lysosomal system and calcium homeostasis, which may cause a selective threat to DA neurons in PD [34].

Similarly to mutations in *GBA1*, mutations in *PINK1* and *PARK2* are also associated with early onset recessive forms of familial PD [45]. Both proteins, PINK1 and Parkin, are involved in the clearance of mitochondrial damage. Therefore their mutations cause a PD characterized by mitochondrial stress as main feature [46,47,48]. Under physiological conditions, Parkin, which is localized in the cytoplasm, is translocated to damaged mitochondria in a PINK-dependent manner triggering mitophagy [49]. This has been confirmed in iPSC-derived DA neurons carrying a mutation in *PINK1*. In these cells, Parkin recruitment to mitochondria was impaired and only over-expression of WT *PINK1* was able to rescue the function [37]. On the other hand, iPSC models for mutation in *PARK2* revealed an increase of oxidative stress. Jiang and colleagues showed that iPSC from patients carrying mutations in *PARK2* enhanced the transcription of monoamine oxidase, the spontaneous release of dopamine and significantly decreased dopamine uptake, increasing susceptibility to reactive oxygen species [35]. Although the incremented oxidative stress has been confirmed in a parallel study, in this study no difference in monoamine oxidase was observed [36]. On the contrary, the oxidative stress was accompanied by a compensation mechanism that involved the activation of the reducing Nrf2A pathway [36].

Mutations in *LRRK2* have been one of the most studied mutations in PD, not only because they are the most common cause of familial PD, but also because clinical symptoms of *LRRK2*-PD are similar to those of idiopathic PD [50]. The most common mutation is the G2019S, which results in hyper-activity of the LRRK2 kinase domain. Although penetrance of this gene has shown to be variable between individuals’ age, iPSC model of a G2019S *LRRK2*-PD has recapitulated characteristic features of PD, such as accumulation of α-syn, increase in genes responsible for oxidative stress and enhanced susceptibility to hydrogen peroxide, which is displayed through caspase-3 activation [39]. Furthermore, the expression of key oxidative stress-response genes and α-syn were found to be increased in neurons from *LRRK2*-iPSC, when compared to those differentiated from control iPSC or hESC.

Our group has generated iPSC lines from seven patients with idiopathic PD and four patients carrying G2019S mutation in the *LRRK2* gene [40]. We observed morphological alterations in PD-derived iPSC vmDAn (fewer and shorter neurites) as well as an increase in the number of apoptotic neurons over a long-time culture (2.5 months). Moreover, we found an accumulation of α-syn in *LRRK2*-iPSC derived DAn after a 30 days culture.

Sporadic forms of PD are not as well defined, given that they may be caused by several genetic variants, as well as a strong environmental effect. However, our study revealed that DAn, which were derived from idiopathic PD patients, also showed an increased susceptibility to degeneration *in vitro* after long-term culture [40].

Importantly, the appearance of the neurodegenerative phenotypes in differentiated DAn from either idiopathic or *LRRK2*-associated PD was shown to be the consequence, at least in part, of impaired autophagy. Blockade of autophagy by lysosomal inhibition showed a specific reduction in autophagic flux by LC3-II immunoblotting, suggesting that the clearance of autophagosomes was compromised [40]. Proteins may also enter the autophagic process directly at the lysosome level, via chaperone-mediated autophagy (CMA). Increased co-localization of α-syn with LAMP2A puncta in iPSC-derived *LRRK2* DAn, revealed a compromised degradation of α-syn by CMA [41]. Although both wild-type and mutant LRRK2 inhibit CMA, G2019S LRRK2 protein was more resistant to the CMA-mediated degradation, resulting in α-syn accumulation [41]. Furthermore, the same phenotype was induced by over-expression of wild-type or G2019S *LRRK2* in control iPSC-derived cultures [40] and rescued by LRRK2 inhibition [42]. Indeed, iPSC-derived DAn cultures from isogenic G2019S *LRRK2* lines (mutation being the sole experimental variable) exhibited an increased mutant-specific apoptosis and decreased neurite outgrowth, as well as alterations in the expression of several pERK (phosphorylated ERK) controlled genes, all of which could be rescued by the inhibition of LRRK2 [42]. Moreover, the genetic correction of LRRK2 mutation resulted in the phenotypic rescue of differentiated neurons with improved neurite length to levels comparable to those of controls.

## 5. Patient-Derived Stem Cells Could Improve Drug Research for PD

An important goal of humanized stem cell-based PD model systems is the screening of potential new drugs that could affect the neurodegenerative process at several levels during its development in specifically affected human cells. Moreover, the availability of such patient-specific stem cell-based model systems could help identifying new pharmacological strategies for the design of personalized therapies. Recently, iPSC-derived forebrain neurons have been used as a platform to screen disease-modifying drugs, highlighting the possibilities of iPSC technology as an *in vitro* cell-based assay system for AD research [51]. A recent study has also taken a significant leap towards personalized medicine for PD patients, by investigating signs of the disease in patient-specific iPSC-derived neurons and testing how the cells respond to drug treatments [38]. The study showed that neurons derived from PD patients carrying mutations in the *PINK1* or *LRRK2* genes display common signs of distress and vulnerability such as abnormalities in mitochondria and increased vulnerability to oxidative stress. However, they found that oxygen consumption rates were lower in cells with mutations in *LRRK2* and higher in cells with the mutations in *PINK1*. Notably, they were able to rescue the phenotype caused by toxins to which the cells were exposed to with various drug treatments, including the antioxidant coenzyme Q10 and rapamycin. Most importantly, the response of iPSC-derived neurons was different depending on the type of familial PD, since drugs that prevented damage to neurons with mutations in *LRRK2,* did not protect neurons with mutations in *PINK1* [38].

In addition, Ryan and colleagues performed a high-throughput screening (HTS) to identify molecules that are capable of protecting DAn from the toxic effect of PD-associated pesticides. They observed that the MEF2-PGC1α pathway contributes to the late-onset PD phenotypes due to the interaction between environmental factors and gene expression [25]. They performed HTS for small molecules capable of targeting the MEF2-PGC1α pathway and they identify isoxazole as new potential therapeutic drug. Isoxazole, not only drove the expression of both MEF2 and PGC1α, but also protected A53T DAn from pesticide-induced apoptosis [25].

Chung and colleagues investigated yeast and iPSC PD models in parallel to discover and reverse phenotypic responses to α-syn. In conjunction to what was previously reported, they showed a connection between α-syn toxicity, accumulation of NO and ER stress [32]. With these results, they took a step further by screening for possible α-syn toxicity suppressors in their iPSC model, to compare with their previous yeast screenings [52,53,54]. In particular they showed that the ubiquitin ligase Nedd4 and its chemical activator NAB2 [53] are able to rescue the α-syn toxicity in patient-derived neurons [32], opening a door to a new potential drug treatment.

These results encourage the use of iPSC technology as a tool to discover potential therapeutic drugs. However, concluding for what recent studies have unveiled up until now focusing only on genetic forms of PD, it remains to be determined whether this advanced technology can be used also in sporadic patients with uncertain genetic cause of the disease.

## 6. Limitations of Using iPSC in Disease Modeling: From Overall Neurodegeneration to the Detailed Mechanisms Involved

### 6.1. Reprogramming and Epigenetic Signatures

Reprogramming increases cell variability due to the introduction of mutations in the genomic DNA [55] and the insertion of exogenous reprogramming genes. Moreover reprogrammed cells maintain a residual DNA methylation signature characteristic of the somatic tissue of origin [56,57,58,59] affecting also gene expression [60]. These issues can affect the predisposition of a given line to differentiate into particular cell type independently of the patient’s genotype, and will abrogate the possibility of using these lines for cell therapy treatment in the future. To decrease the impact of these technical limitations, more than one clone for each iPSC line is usually analyzed. However, the use of integrating methods, such as lenti- and retro-virus infection for gene transduction, not only increases cell variability, but also maintains residual expression of exogenous reprogramming genes that is only partially lost through cell passaging. The residual expression of reprogramming genes can, not only create problems during cell differentiation, but overall iPSC do not need a constant over expression of reprogramming genes. Indeed, the reprogramming process by which a somatic cell acquires pluripotent potential is not a genetic transformation, but an epigenomic one [61], therefore only a transient expression of reprogramming genes needs to be activated. Alternative methods to the retro- or lenti-viral infection, have been recently adopted. These include the use of non-integrating viral vectors such as Sendai virus [62], episomal vectors [63], protein transduction [64], or transfection of modified mRNA transcripts [65]. These methods of reprogramming are relevant in the context of any future clinical applications of iPSCs in the field of transplantable replacement cell therapies.

As aforementioned, one of the major concerns in iPSC modeling through the reprogramming of somatic cells into iPSCs has been that of resetting the identity of these cells back to an embryonic stage, therefore having to consider the generated iPSC-derived neurons as fetal neurons. Given the slow progression of neurodegenerative diseases, the idea of modeling this type of disease in a dish has been highly doubted. However, despite the typical late-onset of PD, the key cellular and molecular pathological mechanisms may have started before the onset of the disease. Therefore, α-syn accumulation, autophagic clearance and mitochondrial dysfunctions, among other pathological mechanisms afforested, could have been active in the early stages of the disease. The cumulative effect of these abnormalities along with the effect of environmental influence, have been shown to progressively encourage neurodegeneration [25]. In addition the use of cell stressors and inducible aging [26] also have shown the possibility of accelerating the appearance of diseased phenotypes in a dish.

### 6.2. Reliable Control Lines and Gene-Editing

Comparative studies require an appropriate control that accounts for differences between lines due only to the genotypic background that exists between individuals. This is especially crucial in diseases whose causative mutations do not have a high penetrance. For example, when complex diseases, such as PD, are modeled with patient- and healthy donor-derived iPSC, the patient iPSC tend to show subtle phenotypes that can be masked by genetic background effects [66]. For this reason, it is imperative to remove the excess genetic variation between iPSC clones and controls, to ensure a more reliable comparative analysis. Given that to obtain iPSC from unaffected siblings or parental controls is not often possible, a solution is to generate isogenic controls directly from the patient iPSCs. In the last years, several research groups have used this approach to correct known mutations [25,26,32,34,42,67], or even utilizing the introduction of the same mutation in control iPSC lines to see the effect of just the mutation itself [42,67]. For this reason, isogenic controls have claimed to be crucial when it comes to assess the impact of any mutation on specific cellular processes. Therefore, editing technologies based on Zinc Fingers Nucleases, TALENs or CRISPR [68], have become indispensable tools in developing comparative studies in iPSC models, allowing for the reduction of iPSC cohorts.

### 6.3. Cell Differentiation and Sorting

**Figure 2:**
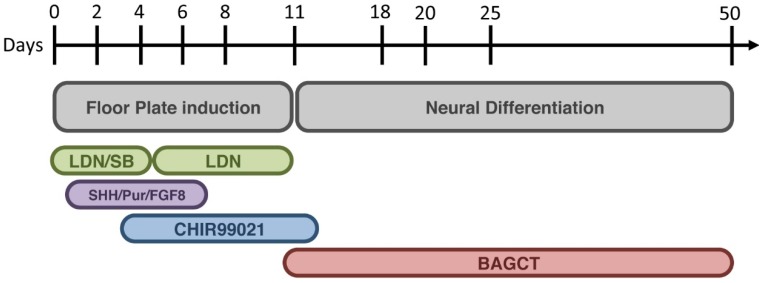
Schematic summary of the novel floor-plate A9 vmDAn differentiation protocol by Kriks [69]. The first stage illustrates floor-plate induction [70], with the appropriate modification in order to reach a more specialized A9 midbrain DA neuronal identity. Exposure to LDN (LDN193189) and SB (SB431542) triggers the Dual-SMAD inhibition. Purmorphamine (Pur), which activates Sonic Hedgehog (SHH) signaling, together with SHH and FGF8 is not sufficient to trigger a selective enrichment of midbrain DA precursors. However, SHH/Pur/FGF8 in combination with exposure to CHIR99021 (a potent GSK3β inhibitor known to strongly activate WNT signaling) allows for a complete enrichment of DA precursors with A9 midbrain identity, by inducing the expression of FOXA2 and LMX1A. Neural differentiation and maturation is achieved through the use of a cocktail of neurotrophic factors BAGCT (BDNF + ascorbic acid + GDNF + dbcAMP + TGFβ3).

The efficacy of Parkinson’s disease iPSC models depends highly on their ability to correctly differentiate neurons into the specific cell type that is affected by the disease (in this case A9 dopaminergic neuronal subtype). Indeed this is critical in order to recapitulate disease features *in vitro* and observe comparative differences between diseased and healthy control lines. Neuronal differentiation of iPSC into DA neurons is not only subjected to high variability of efficiency, depending on the techniques used in a laboratory, but also on the specific ability of each iPSC line. For example, by comparing the studies reported in this review, the percentage of DA neurons compared to the total number of cells varies depending on each cell line, differentiation method and even laboratory group (Table 1). Throughout the field, groups encountered problems in yielding a high percentage of DA neurons within the differentiated population. Therefore, although a number of results are based on the disease phenotype through the identification of TH positive cells by immunocytochemistry, protein immunoblots in which all cell populations are considered skews the data. More specifically, the levels of affected protein in the few TH positive cells may be diluted and missed when mixed with the whole population of differentiate cells when analyzed. Interpretation of these results have been, thus, controversial, especially in the cases in which PD iPSC-derived models have low yield in DA differentiation, which probably cannot go beyond the gross neurodegeneration mechanisms that they have observed. Thus, delving deep inside the biomolecular pathways affected in PD will require a more fine-tuned differentiation protocol that allows the enrichment of the cell type of interest. To achieve this, a novel floor-plate-based strategy described by Kriks and colleagues has become the gold standard in the generation of human A9 vmDA neurons for both transplantation and research purposes [69]. The protocol is based on the concurrent inhibition of two parallel SMAD/TGF-β (transforming growth factor-β) superfamily-signaling pathways, which during CNS development induce no-neuronal fates such as endoderm or mesoderm. This inhibition directs the cell culture to a predetermined neural progenitor fate with an efficiency of at least 80% of PAX6^+^ neural cells among total cells [70]. Differentiation of these neuronal stem cells into mature vmDAn is then instructed through the molecular guidance of Sonic Hedgehog (SHH), FGF8 and more importantly Wnt signaling pathway induction, which enhances expression of the transcription factors FOXA2 and LMX1A [71,72]. The final step of neuronal maturation is achieved through the use of a cocktail of neurotrophic factors, including BDNF, GDNF, TGFβ3, dbcAMP, and ascorbic acid (Figure 2). Interestingly, the most recent papers reviewed here have started to implement the A9 vmDAn enrichment protocol [25,26,34] with the addition of isogenic-corrected controls [25,34]. Moreover, Schöndorf and colleagues improved the Kriks differentiation protocol thanks to the use of a cell sorting method (Fluorescence-activated cell sorting), which allowed for a 6.1-fold enrichment of the neuronal population. This step of sorting was necessary to assess reliable biomolecular changes that could not have been assessed with an unsorted heterogenic population [34].

On the other hand, to unveil the mechanisms behind pathophysiological processes such as neuroinflammation, the investigation of all cells responsible for the maintenance of CNS homeostasis, such as astrocytes and microglia, is crucial. Nevertheless, the study of a more isolated system may allow investigators to detect early events of a disease that would otherwise be missed.

## 7. Conclusions and Challenges

PD is a progressive neurodegenerative disease resulting in the gradual loss of vmDA neurons, as well as cytoplasmic inclusions called Lewy Bodies. The exact mechanisms leading to vmDA neuronal death in PD are still unclear, although pathogenic protein aggregation of α-synuclein, mitochondrial dysfunction, oxidative and nitrosative stress, or altered autophagy have been proposed as mechanisms that contribute to this devastating neurodegenerative process. The generation of reliable iPSC-based models for late-onset neurodegenerative disorders, in which the etiology is yet to be uncovered, has proven to be difficult to overcome. However, recent advances in the field have demonstrated the feasibility of developing experimental models of PD based on iPSC from patients of both genetic and idiopathic forms of PD that recapitulate the key features of the disease. The successful generation of these genetic and idiopathic PD models has opened the door bringing to light some of the crucial pathogenic mechanisms responsible for the initiation and progression of PD, as well as aid in the development of novel drugs that may prevent or rescue neurodegeneration in PD. Recent findings in the field have moved far beyond the proof-of-principle stage, and have started to optimize and standardize these models for the discovery of new aspects of disease biology and new targets for therapeutic intervention. The use of isogenic-corrected controls, more reliable differentiation protocols [25,26,34] and efficient cell-sorting methods [34], have strongly validated the reliability of iPSC models in the context of complex diseases such as PD. Within the field of neuroscience, the opportunity and challenge to combine patient-derived disease-specific stem cells with drug screening technologies with the aim of finding new therapies is now a possibility. In addition, the combination of establishing optimal neuronal differentiation protocols of iPSC using genetic reporters, together with software analysis algorithms, allows for the possibility of automatically tracking each cell over time and to assess any feature of interest, thus providing this system with a powerful tool in drug discovery in the near future.

Moreover, by studying symptomatic and asymptomatic mutation carriers, iPSC technology could also provide a unique opportunity for identifying putative gene-linked PD biomarkers in pre-symptomatic individuals, opening a new novel window for the early diagnosis and individualized treatment in the preclinical phase of the disease.
